# Breaking the cycle: a pilot study on autonomous Digital CBTe for recurrent binge eating

**DOI:** 10.3389/fdgth.2024.1499350

**Published:** 2025-01-17

**Authors:** Rebecca Murphy, Charandeep Khera, Emma L. Osborne

**Affiliations:** Centre for Research on Eating Disorders at Oxford, Department of Psychiatry, Warneford Hospital, University of Oxford, Oxford, United Kingdom

**Keywords:** eating disorders, binge eating, digital, enhanced cognitive behaviour therapy, self-help, smartphone, treatment, online

## Abstract

**Background:**

Only a minority of people with eating disorders receive evidence-based psychological treatment. This is especially true for those with recurrent binge eating because the shame that accompanies binge eating affects help seeking and there is a shortage of therapists to provide psychological treatments. Digital programme-led interventions have the potential to overcome both barriers.

**Objective:**

This study examined the acceptability and effectiveness of a new digital programme-led intervention directly based on enhanced cognitive behaviour therapy (CBT-E), which is an empirically supported psychological treatment for eating disorders.

**Methods:**

One hundred and ten adults with recurrent binge eating (self-reporting characteristics consistent with binge eating disorder, bulimia nervosa, and similar conditions) were recruited through an advertisement on the website of the UK's national eating disorder charity, *Beat*. The intervention, called *Digital CBTe*, comprised 12 sessions over 8–12 weeks delivered autonomously (i.e., without external support). Participants completed self-report outcome measures of eating disorder features and secondary impairment at baseline, post-intervention, and 6-month follow-up.

**Results:**

Most participants identified as female, White, and were living in the United Kingdom. Most participants (85%) self-reported features that resembled binge eating disorder, and the rest self-reported features that resembled bulimia nervosa (8%) and atypical bulimia nervosa (7%). On average, participants reported that the onset of their eating disorder was more than twenty years ago. Sixty-three percent of the participants completed *Digital CBTe* (i.e., completed active treatment sessions). Those who completed all sessions and the post-intervention assessment (*n* = 55, 50%) reported significant decreases in binge eating, eating disorder psychopathology, and secondary impairment at post-intervention. These improvements were maintained at follow-up. Large effect sizes were observed for all these outcomes using a completer analysis and post-intervention data (*d* = 0.91–1.43). Significant improvements were also observed for all outcomes at post-intervention in the intent-to-treat analysis, with medium-to-large effect sizes.

**Discussion:**

A substantial proportion of those who completed *Digital CBTe* and the post-intervention assessment experienced marked improvements. This provides promising data to support the conduct of a fully powered trial to test the clinical and cost-effectiveness of autonomous *Digital CBTe*.

## Introduction

Mental health problems are common and costly to the individuals affected, society, and healthcare services (1–3). Eating disorders are a prime example, as they impact psychosocial functioning and physical health (4–6). The most common eating disorders, bulimia nervosa and binge eating disorder, are characterised by distressing and recurrent episodes of binge eating that occur at least once a week (7, 8). These episodes involve eating unusually large amounts of food accompanied by an aversive sense of being out of control. In bulimia nervosa, binge eating is accompanied by compensatory behaviours, such as self-induced vomiting and laxative misuse. The prevalence rates of bulimia nervosa and binge eating disorder are estimated at 0.8% and 2.2%, respectively (9). Bulimia nervosa and binge eating disorder co-occur with significant psychopathology, mental and physical comorbidity, and life impairment (4, 8, 10).

There is a huge gap between the number of people with eating disorders who could benefit from receiving empirically-supported psychological treatments and those who actually receive them. The first major barrier is that most people with bulimia nervosa or binge eating disorder do not seek help, or delay doing so for many years, primarily due to the shame associated with binge eating (11, 12). People with binge eating disorder have the longest duration of untreated eating disorder in help-seeking populations of any of the eating disorders (13). Even if they do seek help, only a minority of people with eating disorders receive recommended treatments, even in developed countries (7). This is due to the second major barrier: the lack of therapists available to provide treatments (14–16).

Digital programme-led (or self-help) interventions have the potential to overcome both barriers and increase the reach of psychological treatments. Programme-led interventions are those in which the intervention is delivered by the programme itself rather than by a therapist (17). Wider reviews of psychological treatments (17, 18) have concluded that a major change in delivery is required, including capitalising on advances in technology and programme-led interventions, to reach more people. Digital programme-led treatments also offer the potential to reach underserved groups (19).

People with bulimia nervosa or binge eating disorder are well suited to receive programme-led interventions because there is evidence to suggest that they respond to this format of treatment (20, 21). Indeed, UK national guidelines recommend eating-disorder-focused cognitive behavioural self-help—a structured, programme-led approach guided by non-specialists—as the recommended first-line treatment, delivered alongside guidance, for most cases (9). These recommendations are based on rigorous systematic reviews of evidence and expert consensus to ensure they represent the highest standard of care.

The first programme-led treatments for eating disorders were in printed form, but digitalisation of these has the potential to further improve access and increase their potency (17). Furthermore, the COVID-19 pandemic, including measures to prevent it from spreading, has increased risk and symptoms of eating disorders and increased barriers to care (22), increasing the need for early interventions delivered remotely.

Several systematic reviews of digital treatments for eating disorders have been conducted [e.g., (23–25)]. The main conclusions from these reviews are that digital treatment is feasible and acceptable for those with bulimia nervosa and binge eating disorder, and is more effective than waiting list controls in reducing binge eating. It should be noted that there is substantial variability in both completion rates and effectiveness rates for programme-led interventions (26). A recent systematic review and meta-analysis concluded that e-health treatments for eating disorders are more effective than being on a waiting list or receiving information resources, but their efficacy beyond this is unclear and there is heterogeneity across studies (27).

There are several limitations to existing digital treatments for bulimia nervosa and binge eating disorder. First, the digital treatments evaluated in previous research have used some procedures taken from cognitive behavioural therapy (CBT) for binge eating. However, none of the treatments were found to use the full combination and critical sequence recommended in enhanced cognitive behaviour therapy (CBT-E), one of the leading treatments for adults with eating disorders (28). Second, reviews of the features of existing digital interventions for eating disorders have noted their limited technological sophistication and insufficient use of individual tailoring, which is important given that such features improve overall effects (29). Third, much of the research to date has taken place in clinical settings or has tested clinician-supported interventions. There is also a lack of evidence-based apps for eating disorders available outside of healthcare settings in direct-to-user marketplaces (30). Therefore, our research group has developed a new digital treatment that aims to address each of these limitations.

The treatment—called *Digital CBTe*—is a new digital programme-led intervention designed to be the first step in the treatment of bulimia nervosa and binge eating disorder. Its active elements have been directly derived from CBT-E and its associated printed self-help programme, *Overcoming Binge Eating* (31, 32). CBT-E serves as the major exemplar of the broader category of CBT-ED (Cognitive Behavioural Therapy for Eating Disorders), which is recommended by NICE (9). Similarly, Overcoming Binge Eating is the major exemplar of the category of eating-disorder-focused cognitive behavioural self-help programmes, also endorsed by NICE (9), when delivered with guidance from a non-specialist. *Digital CBTe* is designed to be used in a scalable way, including being directly accessed by people in the community and administered through healthcare services with non-specialist support. *Digital CBTe* has been developed and refined on the basis of usability testing and feedback.

The aim of the current pilot study was to assess the preliminary effectiveness of *Digital CBTe* on recurrent binge eating, eating disorder psychopathology, and impairment secondary to the eating disorder.

## Methods

### Design

This was a single-arm pilot intervention study conducted entirely remotely, online, in a real-world community setting. Assessments were carried out at baseline (pre-intervention), post-intervention, and after a 6-month open follow-up period.

### Study population, recruitment, and ethics

Participants were recruited directly from the community through an ad on the website of the UK's national eating disorder charity, *Beat*. The inclusion criteria were self-reported recurrent binge eating over the past three months, and ability to read and write in English. Participants were excluded from the study and directed to more appropriate support if there were risks associated with their participation or if participation could prevent them from receiving another treatment. Exclusion criteria were: aged <18 years old, body mass index (BMI) <18.5 or ≥40.0 (due to potential physical risks and a lack of evidence to support the effectiveness of programme-led treatments in these groups), moderate-to-severe depression or the presence of suicidal thoughts, severe recurrent self-induced vomiting or laxative misuse, co-existing medical condition that influences eating habits, current pregnancy, self-reported substance misuse, and currently receiving or waiting to receive another treatment for binge eating.

We did not perform a power calculation because this study was not designed to test statistically significant impact. The literature provides a range of guidelines for determining sample size in pilot studies, including a rule of thumb of at least 30 participants to estimate a parameter (33). Thirty participants seemed sufficient to meet our objectives of assessing the effects of *Digital CBTe* in this pilot study. Programme-led internet-based interventions are associated with poor retention rates. A recent internet-based cognitive behavioural intervention trial for eating disorder symptoms reported an overall treatment dropout rate of 58% (34). Therefore, our objective was to recruit at least 70 participants.

The Medical Sciences Interdivisional Research Ethics Committee, a subcommittee of the Central University Research Ethics Committee of the University of Oxford, approved the study (No. R61440/RE002). Participants provided their informed consent online through the programme. Post-intervention self-report assessments were also online and integrated into the programme. If these assessments identified a need for additional support, participants were signposted to appropriate resources. *Digital CBTe* was developed by a third-party software firm that passed a security assessment by the University of Oxford. *Digital CBTe* was also subject to penetration testing and vulnerability tests by an external independent agency to ensure the programme's security and resilience.

### Treatment

The intervention, *Digital CBTe*, is described below using recommended reporting guidelines for internet intervention research (35).

#### Focus and target population

*Digital CBTe* is a psychological intervention delivered through smartphone application or website (participants could choose which method to use and switch between them). It targets recurrent binge eating in adults with eating disorders characterised by binge eating (bulimia nervosa, binge eating disorder, and related conditions).

#### Authorship details

*Digital CBTe* was designed by the Centre for Research on Eating Disorders at Oxford (Copyright 2018). All intellectual property is owned by the University of Oxford at the time of the research and the development of *Digital CBTe* was supported by the Wellcome Trust.

#### Model of change

*Digital CBTe* uses a programme-led cognitive-behavioural approach to eating disorders derived from CBT-E (28) and its associated printed self-help programme, *Overcoming Binge Eating* (31, 32).

#### Type and dose of intervention and interactivity

*Digital CBTe* is an automated (type a) treatment intervention for a specific condition, especially well-suited for use as an early intervention. It includes the following: (anonymous) online screening to establish the suitability of the intervention; outcome assessment at baseline and end of treatment; a real time self-monitoring tool and tailoring to an individual's psychopathology (e.g., specific advice on compensatory behaviours if reported). It comprises 12 sessions over 8–12 weeks. Sessions are made available at fixed time intervals, with later sessions having larger gaps between them. *Digital CBTe* was offered as a fully automated (pure “self-help” or “self-guided”) intervention in this study and no additional support or guidance was provided. Participants could email a researcher for asynchronous technical assistance if needed.

### Assessment

People who responded to our advertisement completed a brief self-report suitability screening assessment in *Digital CBTe* to assess inclusion and exclusion criteria.

#### Participant characteristics

The suitability screening assessment included self-report items related to demographics, characteristics of eating disorders, and previous help seeking and treatment experience. People who were eligible were invited to participate and provide informed consent.

#### Treatment completion and outcomes

Treatment completion was defined as the proportion of participants (out of those who consented) who completed the active treatment sessions (i.e., completed session 1–9). This is because sessions 1–9 contain the active treatment ingredients, while sessions 10–12 (“staying well”) help with maintenance and do not introduce any new concepts. In addition to the proportion of participants who completed *Digital CBTe*, the (a) proportion who completed all 12 sessions and (b) average number of sessions completed were reported.

Participants completed online self-report measures at baseline, post-intervention, and 6-month follow-up, to assess:

1. Eating disorder features over the past 28 days—assessed using the Eating Disorder Examination–Questionnaire [EDE-Q; (36)], modified for *Digital CBTe* (see Supplementary Materials). Two outcome variables were created:
  a. A single item that assesses the number of objective binge eating episodes (OBEs) experienced. This was the primary outcome (post-intervention assessment);
  b. The severity of general eating disorder psychopathology (measured by the global EDE-Q score, where 0 is the absence of characteristics and 6 is the most severe).
2. Secondary impairment due to eating disorder features—assessed using the Clinical Impairment Assessment [CIA; (37)].

In line with the optimal procedures used in digital treatment research to minimise missing data, participants were encouraged to complete the outcome assessment (those who completed the post-intervention and 6-month follow-up assessments received a £20 Amazon voucher). It was not possible to obtain outcome data from participants who did not complete all sessions because the post-intervention assessment was part of the final session.

#### Participant view of progress and satisfaction

Participants completed a single item at the end of the final session of *Digital CBTe* that asked about their opinion on whether their binge eating problem had improved. Participants who completed all sessions were also invited to complete a survey on their satisfaction with the programme. The survey contained some items from the Client Satisfaction Questionnaire adapted to internet-based interventions [CSQ-I; (38)] and questions that were specific to *Digital CBTe.* Participants who did not complete all sessions were invited to complete a brief survey asking about their reasons for stopping.

### Data analyses

#### Group outcome analyses

A paired sample *t*-test was used to compare the frequency of binge eating, eating disorder psychopathology, and secondary impairment, from baseline to post-intervention, for participants who completed the post-intervention assessment. Repeated measures analysis of variance (ANOVA) considering scores from each time point, including 6-month follow-up, assessed whether any changes were sustained in the long term. Given the limitations of completer analyses, we also performed a secondary analysis using the last-observation-carried-forward.

#### Clinical significance

A clinical cut-off corresponding to 1 standard deviation (SD) above the mean of a normative sample has been recommended for evaluating clinical significance (39). Consistent with this, we calculated the proportion of participants with a global EDE-Q score <1 SD above the community mean at each time point [i.e., <2.77; (40)] to effectively track improvement in eating disorder features through treatment and follow-up.

## Results

### Study participants

#### Recruitment and retention

**Figure 1:**
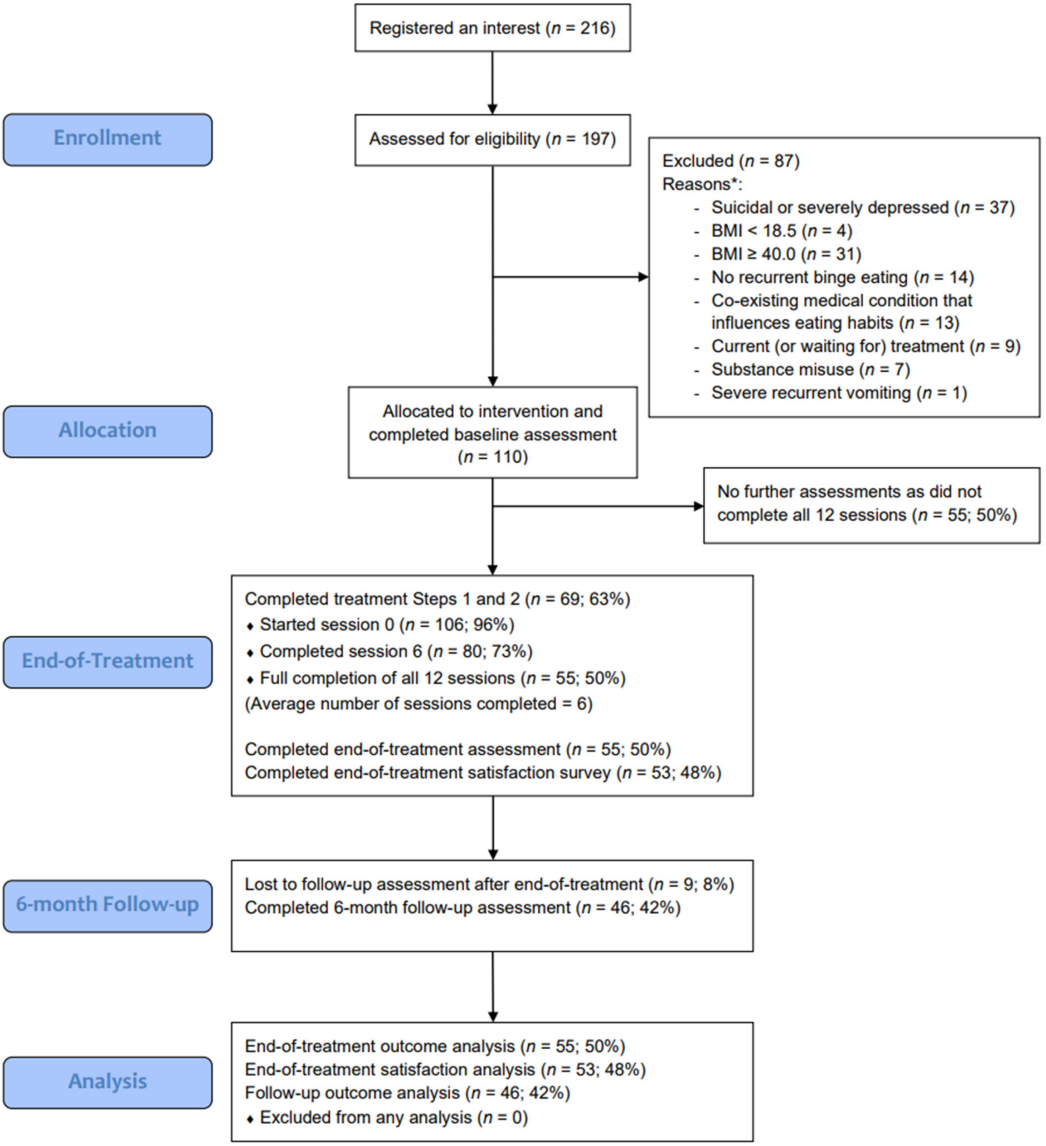
CONSORT flow diagram. *Some participants had multiple reasons for exclusion.

One hundred and ten people were recruited. Figure 1 shows the CONSORT flow diagram.

#### Demographic, eating disorder and help-seeking characteristics

Table 1 shows the characteristics of the sample at baseline. The mean age was 40 years and most of the participants identified themselves as female, White, and resident in the United Kingdom. On average, participants reported (objective) binge eating every other day and that the onset of their eating disorder was more than twenty years ago.

**Table 1:** Characteristics of the sample at baseline (*N* = 110).

Variable	*M (SD)*	Range
Age (years)	39.7 (10.9)	20–63
BMI (kg/m^2^)	27.7 (5.3)	18.1^a^–39.2
Years since onset of eating disorder	22.8 (12.6)	1–51
Frequency of objective binge eating (over the past four weeks)	14.1 (8.1)	4–40
Eating disorder psychopathology (Global EDE-Q)	3.7 (0.9)	1.5–5.6
Secondary impairment (CIA)	28.0 (8.9)	2–48
	*N* (%)	
Female	102 (92.7)	
Male	8 (7.3)	
Ethnicity		
White	102 (92.7)	
Asian	3 (2.7)	
Black	1 (0.9)	
Mixed	1 (0.9)	
Prefer not to say	3 (2.7)	
Country of residence		
United Kingdom	80 (72.7)	
United States	15 (13.6)	
Canada	5 (4.5)	
Australia	4 (3.6)	
New Zealand	4 (3.6)	
Republic of Ireland	2 (1.8)	

^a^ One participant had a BMI <18.5 at baseline (18.1); however, this participant had BMI >18.5 at eligibility screening (18.8). This participant was eligible, but there were slight changes in the height and weight reported at baseline (up to two weeks after completing the eligibility questionnaire).

Using the EDE-Q, the following diagnostic subgroups were created:

• Self-reported features that resembled binge eating disorder (*n* = 93, 85%). This was operationalised as at least four objective binge eating episodes and the absence of purging behaviour (self-induced vomiting or laxative misuse) in the past 28 days.
• Self-reported features that resembled bulimia nervosa (*n* = 9, 8%). This was operationalised as at least four objective binge eating episodes and at least four episodes of purging behaviour (self-induced vomiting or laxative misuse) in the past 28 days.
• Self-reported features that resembled atypical bulimic disorder (*n* = 8, 7%). This was operationalised as at least four objective binge eating episodes in the past 28 days and between 1 and 3 episodes of purging behaviour (self-induced vomiting or laxative misuse) in the past 28 days.

Three-quarters of participants (*n* = 82) reported that they had not received prior treatment for their eating disorder. Within this group, in response to closed-ended questions with pre-defined response options, 65% (*n* = 53) had wanted help with their eating problem in the past but had been discouraged from seeking help due to “shame” (*n* = 40, 75%) and “not knowing there were effective treatments” (*n* = 34, 64%). More than half of this group who had wanted help in the past (*n* = 30, 57%) reported having been told that “there was no one in your local area able to treat your problem” (*n* = 16, 53%), “there was a long waiting list for treatment” (*n* = 11, 37%), and “your problem was not severe enough” (*n* = 15, 50%).

### Treatment completion and outcomes

#### Completion

Sixty-three percent of participants (*n* = 69) completed *Digital CBTe* (i.e., completed active treatment sessions), and 50% (*n* = 55) completed all sessions. The average number of sessions completed across participants who consented (*n* = 110) was six.

#### Outcomes

**Figure 2:**
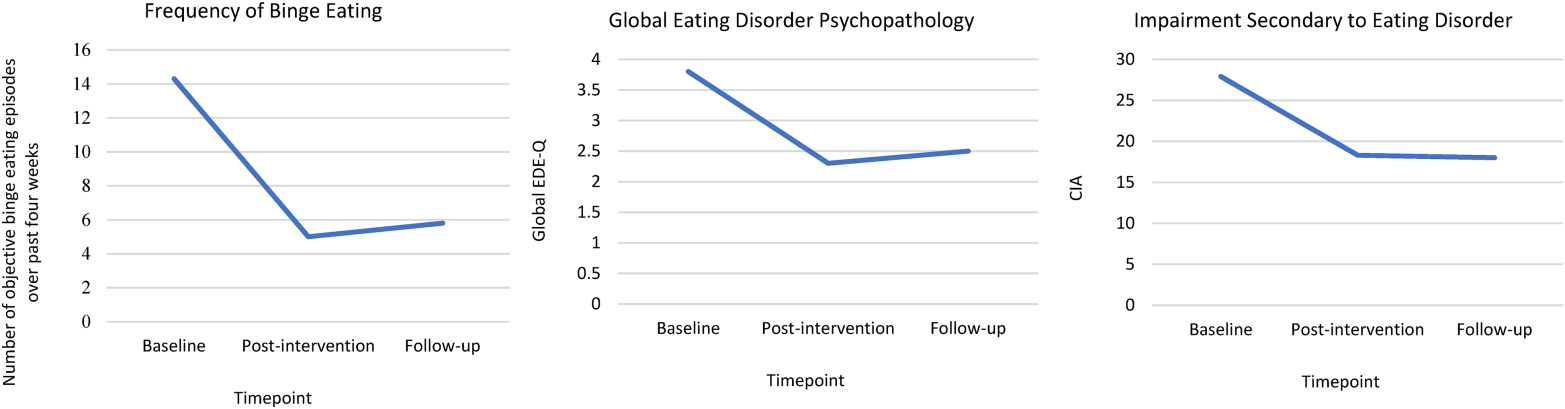
Outcomes at baseline, post-intervention, and at 6-month follow-up for participants who completed the post-intervention and follow-up assessments.

Significant pre-post improvements in the frequency of objective binge eating episodes (primary outcome), eating disorder psychopathology, and secondary impairment (secondary outcomes) were observed, with large effect sizes (Table 2). These improvements were maintained at 6-month follow-up (Figure 2 and Supplementary Table S1).

**Table 2:** Differences in outcomes between baseline and post-intervention for participants who completed the post-intervention assessment (*n* = 55).

Variable	Baseline		Post-intervention		M_diff_ (*SD*)	*t*-test (*df* = 54)	*p*	Cohen *d*
	*M*	*SD*	*M*	*SD*				
Frequency of objective binge eating (over past four weeks)^a^	14.9	8.1	5.5	5.1	9.4 (8.5)	8.20	<.001	1.11
Eating disorder psychopathology (Global EDE-Q)	3.7	0.9	2.3	0.9	1.4 (1.0)	10.59	<.001	1.43
Secondary impairment (CIA)	27.5	9.2	18.2	10.4	9.3 (10.2)	6.74	<.001	0.91

^a^ We report descriptive statistics for the untransformed variable and used the log(x + 1) transformed variable for purpose of analysis.

#### Clinical significance

Sixty-nine percent (*n* = 38) of participants who completed the post-intervention assessment experienced a clinically significant improvement [i.e., had a global EDE-Q score <1 SD above the community mean at post-intervention (40)].

#### Sensitivity analysis

Using last-observation-carried-forward to manage missing data for those who did not complete the post-intervention assessment, we found significant reductions in the frequency of objective binge eating episodes, eating disorder psychopathology, and secondary impairment, from baseline to post-intervention (Supplementary Table S2). Effect sizes were medium-large.

#### Participant view of progress and satisfaction

Of those who completed the “view of progress” question in the final session of *Digital CBTe*, 95% (*n* = 53) reported that their binge eating problem was at least a bit better. Specifically, 21% (*n* = 12) said “my binge eating problem is much better”, 39% (*n* = 22) said “my binge eating problem is somewhat better”, 34% (*n* = 19) said “my binge eating problem is a bit better”, and 5% (*n* = 3) said “my binge eating problem isn't any better”.

Of those who completed the satisfaction survey (*n* = 53, 48% of the total sample), three quarters reported finding *Digital CBTe* “very helpful” (*n* = 22, 42%) or “moderately helpful” (*n* = 18, 34%) with the remainder finding it “somewhat helpful” (*n* = 13, 25%). Almost all participants reported that they would “definitely recommend” (*n* = 31, 58%) or “possibly recommend” (*n* = 19, 36%) *Digital CBTe* to someone else who has a binge eating problem. Together, these findings suggest that most people who completed all 12 sessions were satisfied with *Digital CBTe*. Only 10 participants (18% of those who did not complete all 12 sessions of *Digital CBTe*) completed the “Stopping Digital CBTe” questionnaire. The most commonly selected pre-defined response option for stopping was “*Digital CBTe* was not helping me” (*n* = 5, 50%). Free text responses included the research study being at an inconvenient time, struggling with technical issues, and wishing that the programme was more tailored to their particular needs.

## Discussion

### Main findings and implications

In this preliminary study of the effectiveness of a digital intervention for recurrent binge eating, delivered directly to users and used autonomously, just under two-thirds of the participants completed *Digital CBTe* (i.e., completed active treatment sessions), and half completed all sessions (including maintenance sessions) over 8–12 weeks. A substantial proportion of participants who completed all sessions reported substantial benefit. The effects were not restricted to binge eating; improvements in general eating disorder psychopathology and secondary impairment were also found. This was despite the participants having a longstanding eating problem. These effects were also maintained at 6-month follow-up.

This study represents a significant step forward as the first to evaluate the preliminary effects of a novel intervention: the digital, programme-led version of CBT-E. Although this method of delivery is novel, the CBT-E treatment model is well established with strong empirical support. These findings are of particular importance given this new method of reaching and treating people with binge eating, i.e., direct-to-user and without the need for additional support.

This digital self-help programme is ideally suited to treat binge eating, given the high levels of shame associated with this problem (11, 12). It allows for people to be treated outside of traditional healthcare settings, at a time and place convenient to them. Participants can also use the intervention without requiring a formal diagnosis, relying on self-reported features and self-identified need, empowering them to address their mental health proactively. It therefore has the potential for massive scale and reach, providing access to large numbers of people, including underserved groups, at relatively low cost. Typically, healthcare services are only able to treat the “tip of the iceberg” (i.e., the seen minority) in terms of the scale of eating problems. Scalability is crucial given the large treatment gap and limitations of traditional care (16).

It is notable that the reports from participants taking part in this study were entirely consistent with the benefits of offering treatment direct-to-user i.e., a large proportion had either sought help but were unable to access it or had not sought help due to shame. It was also of interest that the sample was mainly characterised by individuals who reported the onset of their eating problem over 20 years ago. This may be because the study mainly attracted people who were urgently seeking treatment, as the participants were recruited through an eating disorder charity.

Although the completion rate in this study exceeded the average rate of 36% reported for e-health interventions targeting eating disorders (27), the observed dropout rate still reflects the broader issue of suboptimal completion in digital interventions. This is concerning because high adherence—defined as consistent engagement with the intervention as prescribed—and completion—finishing all treatment components—are associated with better outcomes [e.g., (41)]. Therefore, improving adherence and completion should be a critical focus of future research to optimise the effectiveness of this intervention.

### Study strengths and limitations

A key strength of this study was its setting, which intentionally limited participant interaction with researchers and conducted the intervention outside of a clinic. By doing so, the study minimised potential expectation effects—biases that can arise when participants alter their behaviour or responses based on perceived researcher expectations or the clinical environment. These design choices were carefully implemented to ensure the intervention was evaluated under real-world conditions, reflecting how it would be used in practice (42). This approach enhances the ecological validity of the findings and supports the scalability and applicability of the intervention in non-clinical settings.

In terms of study limitations, the sample of participants was predominantly White and female. Therefore, firstly, it cannot be assumed that the promising effects would extend to other groups, and secondly, it needs to be considered whether *Digital CBTe* could engage and help a more diverse group of people. This is important given the evidence that eating disorders affect ethnic minority groups as much as White ethnic groups (43), yet most of the participants in eating disorder research are white (44). Furthermore, outcome data were unavailable for participants who did not complete all sessions, which may introduce bias in the results. Dropout is most likely associated with not benefiting from the programme, potentially overestimating the effectiveness of the intervention and limiting the generalisability of the findings. To attempt to address this, an intent-to-treat analysis was conducted with last observation carried forward to partially mitigate the impact of this missing data. Notably, this analysis was consistent with our previous findings. Future research should aim to collect data from all participants, including those who do not complete all sessions.

A key limitation was the absence of a comparison arm, making it difficult to confirm whether the observed effects were due to the programme or other factors, such as spontaneous remission. While spontaneous remission is less likely over the brief treatment duration, incorporating a comparison arm in future research would strengthen the evidence for the efficacy of *Digital CBTe*. Another limitation was that participants were not restricted from accessing other treatments during the 12-week study. However, many participants reported being unable to access help elsewhere prior to starting the programme, suggesting that the impact of concurrent treatments may have been minimal. To better isolate the effects of Digital CBTe, future studies should collect detailed data on concurrent treatments or consider adopting a closed-treatment design.

### Future directions

There are several future directions. First, to consider how to improve and assess the “reach” of *Digital CBTe* given its huge potential in this area. This will include an examination of what proportion of people who learn about *Digital CBTe* would consider using it and, of these, what proportion would engage with and start the treatment. It will also require an evaluation of the demographic and eating disorder characteristics of the users to ensure that the intervention reaches a wide spectrum of individuals.

Second, while the completion rate of 69% in our study was higher than the average for e-health interventions for eating disorders [36%; (27)], it remains necessary to improve this further. Strategies such as incorporating “support” or “guidance” could enhance adherence (45), but such measures are likely to compromise scalability. Exploring scalable and innovative solutions, such as generative artificial intelligence and large language models, may provide promising alternatives (46).

Third, these preliminary findings require replication and extension. The next step would be a controlled clinical trial to establish the clinical and cost effectiveness of *Digital CBTe*. Given that it remains unclear how, for whom, and under what circumstances digital eating disorder programmes are effective (47) future research should focus on conducting more high-quality randomised controlled trials to explore the participant and intervention characteristics that predict, mediate and moderate treatment success (48). Therefore, such a future trial should be used to predictors, mediators and moderators of outcome for *Digital CBTe*. This could help target the programme to those who are most likely to benefit from the intervention and potentially improve its potency.

However, identifying robust moderators or mediators has typically been fraught with challenges, many of which have been attributed to methodological problems, and overcoming these requires a thoughtful combination of exploratory and confirmatory approaches (49). It has also been argued that even if these methodological problems were addressed, that mediators and moderators will remain elusive unless temporal context is adequately considered—ensuring interventions are delivered at “pivotal moments” when individuals need them most (50). These moments could include initial screening, adaptive delivery during moments of need throughout treatment, or post-discharge.

Finally, collecting data and feedback from those who do not complete all sessions of *Digital CBTe* is vital. Understanding the reasons for disengagement can inform programme modifications and help design an intervention that better meets the needs of a wider sample.

## Conclusions

This preliminary effectiveness study found that *Digital CBTe* offered substantial benefits to people with recurrent binge eating who completed all sessions of the intervention. These effects were maintained after treatment and extended beyond binge eating to more general eating disorder psychopathology and impairment. *Digital CBTe* was delivered in a highly scalable format: autonomously, without additional support and directly to users (outside of healthcare). *Digital CBTe* could be a “game changer” by making it possible to offer an evidence-based treatment to the vast majority of binge eating issues that often go unnoticed, much like the hidden nine-tenths of an iceberg. Notwithstanding these promising findings, crucial next steps would include improving completion rates for *Digital CBTe* and conducting a clinical trial to evaluate its clinical and cost-effectiveness.

## Data Availability

The datasets generated during this study are not publicly available due to privacy protection concerns. Requests to access the datasets should be directed to the corresponding author.

## References

[1] Arias D Saxena S Verguet S. Quantifying the global burden of mental disorders and their economic value. EClinicalMedicine. (2022) 54:1–10. 10.1016/j.eclinm.2022.101675 PMC9526145 36193171

[2] Vigo D Thornicroft G Atun R. Estimating the true global burden of mental illness. Lancet Psychiatry. (2016) 3(2):171–8. 10.1016/S2215-0366(15)00505-2 26851330

[3] Whiteford HA Degenhardt L Rehm J Baxter AJ Ferrari AJ Erskine HE Global burden of disease attributable to mental and substance use disorders: findings from the global burden of disease study 2010. Lancet. (2013) 382(9904):1575–86. 10.1016/S0140-6736(13)61611-6 23993280

[4] Ágh T Kovács G Pawaskar M Supina D Inotai A Vokó Z. Epidemiology, health-related quality of life and economic burden of binge eating disorder: a systematic literature review. Eat Weight Disord. (2015) 20(1):1–12. 10.1007/s40519-014-0173-9 25571885 PMC4349998

[5] Ahmed M Islam MD Aouad P Miskovic-Wheatley J Touyz S Maguire S Global and regional economic burden of eating disorders: a systematic review and critique of methods. Int J Eating Disord. (2024):1–26. 10.1002/eat.24302 PMC11784850 39542867

[6] Mitchison D Hay P Slewa-Younan S Mond J. Time trends in population prevalence of eating disorder behaviors and their relationship to quality of life. PLoS One. (2012) 7(11):e48450. 10.1371/journal.pone.0048450 23144886 PMC3492397

[7] American Psychiatric Association. Diagnostic and Statistical Manual of Mental Disorders. 5th ed. Arlington, VA: American Psychiatric Publishing (2013).

[8] Kessler RC Berglund PA Chiu WT Deitz AC Hudson JI Shahly V The prevalence and correlates of binge eating disorder in the world health organization world mental health surveys. Biol Psychiatry. (2013) 73(9):904–14. 10.1016/j.biopsych.2012.11.020 23290497 PMC3628997

[9] National Institute for Health and Care Excellence. Eating disorders: recognition and treatment (NICE Guideline NG69). (2020). Available online at: https://www.nice.org.uk/guidance/ng69 (Accessed December 19, 2024). 39405396

[10] Hilbert A. Binge-eating disorder. Psychiatr Clin. (2019) 42(1):33–43. 10.1016/j.psc.2018.10.011 30704638

[11] Strandskov SW Ghaderi A Andersson H Parmskog N Hjort E Wärn AS Effects of tailored and ACT-influenced internet-based CBT for eating disorders and the relation between knowledge acquisition and outcome: a randomized controlled trial. Behav Ther. (2017) 48(5):624–37. 10.1016/j.beth.2017.02.002 28711113

[12] Wagner B Nagl M Dölemeyer R Klinitzke G Steinig J Hilbert A Randomized controlled trial of an internet-based cognitive-behavioral treatment program for binge-eating disorder. Behav Ther. (2016) 47(4):500–14. 10.1016/j.beth.2016.01.006 27423166

[13] Austin A Flynn M Richards K Hodsoll J Antunes T Paul D Duration of untreated eating disorder and relationship to outcomes: a systematic review of the literature. Eur Eat Disord Rev. (2020) 29(3):1–17. 10.1002/erv.2745 32578311

[14] Fairburn CG Patel V. The global dissemination of psychological treatments: a road map for research and practice. Am J Psychiatry. (2014) 171(5):495–8. 10.1176/appi.ajp.2013.13111546 24788281

[15] Fairburn CG Patel V. The impact of digital technology on psychological treatments and their dissemination. Behav Res Ther. (2017) 88:19–25. 10.1016/j.brat.2016.08.012 28110672 PMC5214969

[16] Kazdin AE Fitzsimmons-Craft EE Wilfley DE. Addressing critical gaps in the treatment of eating disorders. Int J Eating Disord. (2017) 50(3):170–89. 10.1002/eat.22670 PMC6169314 28102908

[17] Singla DR Kohrt BA Murray LK Anand A Chorpita BF Patel V. Psychological treatments for the world: lessons from low-and middle-income countries. Annu Rev Clin Psychol. (2017) 13(1):149–81. 10.1146/annurev-clinpsy-032816-045217 28482687 PMC5506549

[18] Kazdin AE Blase SL. Rebooting psychotherapy research and practice to reduce the burden of mental illness. Perspect Psychol Sci. (2011) 6(1):21–37. 10.1177/1745691610393527 26162113

[19] Aardoom JJ Dingemans AE Van Furth EF. E-Health interventions for eating disorders: emerging findings, issues, and opportunities. Curr Psychiatry Rep. (2016) 18(4):1–8. 10.1007/s11920-016-0673-6 26946513

[20] De Zwaan M Herpertz S Zipfel S Svaldi J Friederich HC Schmidt F Effect of internet-based guided self-help vs individual face-to-face treatment on full or subsyndromal binge eating disorder in overweight or obese patients: the INTERBED randomized clinical trial. JAMA Psychiatry. (2017) 74(10):987–95. 10.1001/jamapsychiatry.2017.2150 28768334 PMC5710472

[21] Ter Huurne ED De Haan HA Postel MG Van Der Palen J Van Der Nagel JEL DeJong CAJ. Web-based cognitive behavioral therapy for female patients with eating disorders: randomized controlled trial. J Med Internet Res. (2015) 17(6):e152. 10.2196/jmir.3946 26088580 PMC4526949

[22] Rodgers RF Lombardo C Cerolini S Franko DL Omori M Fuller-Tyszkiewicz M The impact of the COVID-19 pandemic on eating disorder risk and symptoms. Int J Eating Disord. (2020) 53(7):1166–70. 10.1002/eat.23318 PMC7300468 32476175

[23] Aardoom JJ Dingemans AE Spinhoven P Van Furth EF. Treating eating disorders over the internet: a systematic review and future research directions. Int J Eating Disord. (2013) 46(6):539–52. 10.1002/eat.22135 23674367

[24] Dölemeyer R Tietjen A Kersting A Wagner B. Internet-based interventions for eating disorders in adults: a systematic review. BMC Psychiatry. (2013) 13(6):207. 10.1186/1471-244X-13-207 23919625 PMC3750530

[25] Melioli T Bauer S Franko DL Moessner M Ozer F Chabrol H Reducing eating disorder symptoms and risk factors using the internet: a meta-analytic review. Int J Eating Disord. (2016) 49(1):19–31. 10.1002/eat.22477 26607683

[26] Sysko R Walsh BT. A critical evaluation of the efficacy of self-help interventions for the treatment of bulimia nervosa and binge-eating disorder. Int J Eating Disord. (2008) 41(2):97–112. 10.1002/eat.20475 17922533

[27] Linardon J Shatte A Messer M Firth J Fuller-Tyszkiewicz M. E-mental health interventions for the treatment and prevention of eating disorders: an updated systematic review and meta-analysis. J Consult Clin Psychol. (2020) 88(11):994–1007. 10.1037/ccp0000575 32852971

[28] Fairburn CG. Cognitive Behavior Therapy and Eating Disorders. New York, NY: Guilford Press (2008).

[29] Barakat S Maguire S Smith KE Mason TB Crosby RD Touyz S. Evaluating the role of digital intervention design in treatment outcomes and adherence to eTherapy programs for eating disorders: a systematic review and meta-analysis. Int J Eating Disord. (2019) 52(10):1077–94. 10.1002/eat.23131 31328815

[30] O’Leary T Torous J. Smartphone apps for eating disorders: an overview of the marketplace and research trends. Int J Eating Disord. (2022) 55(5):625–32. 10.1002/eat.23690 35175640

[31] Fairburn CG. Overcoming Binge Eating. New York, NY: Guilford Press (1995).

[32] Fairburn CG. Overcoming Binge Eating. 2nd ed. New York, NY: Guilford Press (2013).

[33] Browne RH. On the use of a pilot sample for sample size determination. Stat Med. (1995) 14(17):1933–40. 10.1002/sim.4780141709 8532986

[34] Linardon J Messer M Shatte A Greenwood CJ Rosato J Rathgen A Does the method of content delivery matter? Randomized controlled comparison of an internet-based intervention for eating disorder symptoms with and without interactive functionality. Behav Ther. (2022) 53(3):508–20. 10.1016/j.beth.2021.12.001 35473653

[35] Proudfoot J Klein B Barak A Carlbring P Cuijpers P Lange A Establishing guidelines for executing and reporting internet intervention research. Cogn Behav Ther. (2011) 40(2):82–97. 10.1080/16506073.2011.573807 25155812

[36] Fairburn CG Beglin SJ. Assessment of eating disorders: interview or self-report questionnaire? Int J Eating Disord. (1994) 16(4):363–70. 10.1002/1098-108X(199412)16:4<363::AID-EAT2260160405>3.0.CO;2-# 7866415

[37] Bohn K Doll HA Cooper Z O’Connor M Palmer RL Fairburn CG. The measurement of impairment due to eating disorder psychopathology. Behav Res Ther. (2008) 46(10):1105–10. 10.1016/j.brat.2008.06.012 18710699 PMC2764385

[38] Bo L Lehr D Reis D Vis C Riper H Berking M Reliability and validity of assessing user satisfaction with web-based health interventions. J Med Internet Res. (2016) 18(8):1–13. 10.2196/jmir.5952 PMC5023944 27582341

[39] Kendall PC Marrs-Garcia A Nath SR Sheldrick RC. Normative comparisons for the evaluation of clinical significance. J Consult Clin Psychol. (1999) 67(3):285–99. 10.1037/0022-006X.67.3.285 10369049

[40] Mond JM Hay PJ Rodgers B Owen C. Eating disorder examination questionnaire (EDE-Q): norms for young adult women. Behav Res Ther. (2006) 44(1):53–62. 10.1016/j.brat.2004.12.003 16301014

[41] Donkin L Christensen H Naismith SL Neal B Hickie IB Glozier N. A systematic review of the impact of adherence on the effectiveness of e-therapies. J Med Internet Res. (2011) 13(3):e52. 10.2196/jmir.1772 21821503 PMC3222162

[42] Fitzsimmons-Craft EE. Thinking flexibly about who digital mental health interventions are for and how they should be evaluated and used: commentary on McClure et al. (2023). Int J Eat Disord. (2024) 57(5):1130–3. 10.1002/eat.24136 38180103 PMC11093696

[43] Cheng ZH Perko VL Fuller-Marashi L Gau JM Stice E. Ethnic differences in eating disorder prevalence, risk factors, and predictive effects of risk factors among young women. Eat Behav. (2019) 32:23–30. 10.1016/j.eatbeh.2018.11.004 30529736 PMC6382562

[44] Egbert AH Hunt RA Williams KL Burke NL Mathis KJ. Reporting racial and ethnic diversity in eating disorder research over the past 20 years. Int J Eating Disord. (2022) 55(4):455–62. 10.1002/eat.23666 PMC11389655 34997609

[45] Musiat P Johnson C Atkinson M Wilksch S Wade T. Impact of guidance on intervention adherence in computerised interventions for mental health problems: a meta-analysis. Psychol Med. (2022) 52(2):229–40. 10.1017/S0033291721004621 34802474

[46] Ramos G Hernandez-Ramos R Taylor M Schueller SM. State of the science: using digital mental health interventions to extend the impact of psychological services. Behav Ther. (2024) 55(6):1364–79. 10.1016/j.beth.2024.04.004 39443071

[47] McClure Z Fuller-Tyszkiewicz M Messer M Linardon J. Predictors, mediators, and moderators of response to digital interventions for eating disorders: a systematic review. Int J Eat Disord. (2024) 57(5):1034–48. 10.1002/eat.24078 37886906

[48] Bauer S Moessner M. We need to be more critical toward digital interventions for eating disorders: a commentary on McClure et al. (2023). Int J Eat Disord. (2024) 57(5):1123–5. 10.1002/eat.24119 38126231

[49] Kraemer HC. Envisioning an improved research strategy for detecting moderators/mediators in intervention studies: a commentary on McClure et al. Int J Eat Disord. (2024) 57(5):1138–40. 10.1002/eat.24157 38353420

[50] Schleider JL Smith AC Graham AK. Timing matters in (mis)identifying moderators and mediators of digital interventions for eating disorders: commentary on McClure et al. (2023). Int J Eat Disord. (2024) 57(5):1141–4. 10.1002/eat.24185 38450821 PMC11093699

