# Supplementary material for: Breaking the cycle: a pilot study on autonomous Digital CBTe for recurrent binge eating
Source: Front Digit Health. 2025 Jan 17;6:1499350. doi: 10.3389/fdgth.2024.1499350 (PMC11782557; doi:10.3389/fdgth.2024.1499350)
Supplement: Supplementary file 1 [file Datasheet1.pdf]

Breaking the Cycle: A Pilot Study on Autonomous Digital CBTe for Recurrent Binge Eating

Supplementary Materials

EDE-Q Modified for Digital CBTe

The modification to the Eating Disorder Examination–Questionnaire (EDE-Q; Fairburn & Beglin, 2008) in Digital CBTe does not affect calculation of the EDE-Q global and subscale scores. The wording of questions 13, 14, and 15 in the EDE-Q in Digital CBTe have been modified. The reason for this is to include a measure of subjective binge eating, which refers to episodes of loss of control over eating when the amount of food consumed is not objectively large. The original EDE-Q does not tell us about subjective binge eating; however, the modifications to questions 13–15 tell us about frequency of subjective binge eating without additional burden.

Secondary analysis results

Repeated measures ANOVAs (time: baseline, post-intervention, 6-month follow-up) indicated that frequency of binge eating, eating disorder psychopathology, and secondary impairment, differed between timepoints (see Table S1). Post hoc analyses with a Bonferroni adjustments indicated that: frequency of binge eating decreased from baseline to post-intervention (9.3, 95% CI [6.4, 12.2], $p < .001$), and from baseline to follow-up (8.5, 95% CI [5.5, 11.5], $p < .001$), but not from post-intervention to follow-up (-0.8, 95% CI [-3.1, 1.5], $p = 1.00$); eating disorder psychopathology decreased from baseline to post-intervention (1.4, 95% CI [1.1, 1.8], $p < .001$), and from baseline to follow-up (1.2, 95% CI [0.8, 1.7], $p < .001$), but not from post-intervention to follow-up (-0.2, 95% CI [-0.5, 0.2], $p = 0.775$); secondary impairment decreased from baseline to post-intervention (9.6, 95% CI [5.7, 13.5], $p < .001$), and from baseline to follow-up (9.9, 95% CI [5.2, 14.6], $p < .001$), but not from post-intervention to follow-up (0.3, 95% CI [-3.4, 4.1], $p = 1.00$).

Table S1

Secondary analysis: Differences in outcomes between baseline, post-intervention, and follow-up for participants who were present at follow-up

	Baseline		Post-Intervention		Follow-up		*F*	*df*	*p*	Partial eta^2
Variable	*M*	*SD*	*M*	*SD*	*M*	*SD*				
Frequency of binge eating (over past four weeks)[a]	14.3	7.7	5.0	4.5	5.8	6.6	41.81	2, 90	<.001	0.48
Eating disorder psychopathology (Global EDE-Q)	3.8	0.9	2.3	0.9	2.5	1.2	52.43	2, 90	<.001	0.54
Secondary impairment (CIA)	27.9	9.6	18.3	10.9	18.0	12.2	22.94	2, 88	<.001	0.34

Note. *n* = 46 for frequency of binge eating and eating disorder psychopathology, and *n* = 45 for secondary impairment.

[a] We report descriptive statistics for the untransformed variable and use the log(x+1) transformed variable for purpose of analysis.

Table S2

Sensitivity analysis: Differences in outcomes between baseline and post-intervention with baseline scores carried forward for people who did not complete the intervention (*N* =110)

	Baseline		Post-Intervention		MD[b]	SED[c]	*t* test (*df*=109)	*p*	Cohen's d
	M	*SD*	*M*	*SD*					
Frequency of binge eating (over past four weeks)[a]	14.1	8.1	9.4	7.8	4.7	0.7	6.48	<.001	0.62
Eating disorder psychopathology (Global EDE-Q)	3.7	0.9	3.0	1.1	0.7	0.09	7.45	<.001	0.71
Secondary impairment (CIA)	28.0	8.9	23.4	10.9	4.7	8.6	5.68	<.001	0.54

[a] We report descriptive statistics for the untransformed variable and use the log(x+1) transformed variable for purpose of analysis.

[b] MD = Mean Difference.

[c] SED = Standard Error Difference
